# Supplementary material for: Psychometric evaluation of an Italian custom 4-item short form of the PROMIS anxiety item bank in immune-mediated inflammatory diseases: an item response theory analysis
Source: PeerJ. 2021 Oct 27;9:e12100. doi: 10.7717/peerj.12100 (PMC8556715; doi:10.7717/peerj.12100)
Supplement: Supplemental Information 1 [file peerj-09-12100-s001.docx]

**Appendix A**

Items of the Italian custom 4-item short form adaptation of the PROMIS anxiety short form 8a

Negli ultimi 7 giorni …

1) Mi sono sentito/a a disagio

2) Mi sono sentito/a nervoso/a

3) Mi sono sentito/a ansioso/a

4) Mi sono sentito/a teso/a
